# Effect of Protease Combined with Heat Treatment on the Volatile Composition and Aroma Quality in Liqueur Wine

**DOI:** 10.3390/molecules28135129

**Published:** 2023-06-30

**Authors:** Wen Li, Zhen Zhang, Yuanyuan Zhao, Wei Li, Li Wang, Qi Shang, Jianming Du, Lina Jin

**Affiliations:** College of Food Science and Engineering, Gansu Agricultural University, Lanzhou 730070, China

**Keywords:** liqueur wine, protease, HS-SPME-GC/MS, aroma quality, OPLS-DA

## Abstract

The aim of this paper was to compare the effects of two clarification methods, protease combined with heat treatment and bentonite, on the aroma quality of liqueur wines, and to identify and analyze the overall differences between the basic components and volatile aroma compounds of liqueur wines after the two treatments by chemical analysis, headspace–solid-phase microextraction–gas chromatography/mass spectrometry (HS-SPME-GC/MS), and orthogonal partial least squares discriminant analysis (OPLS-DA). The results showed that total acidity, volatile acidity and pH in liqueur wines after protease combined with heat treatment were not significantly different from those of the blank control, and the ability to remove proteins was equal to that of the bentonite treatment. A total of 58 volatile aroma compounds were detected by HS-SPME-GC/MS. Compared with the blank control group (44 species, total 108.705 mg/L), 52 (83.233 mg/L) and 50 (120.655 mg/L) aroma compounds were detected in the bentonite and protease combined with heat treatments, respectively. Compared with the control and bentonite treatment, the protease combined with heat treatment significantly increased the total content of aromatic compounds in liqueur wines, and the types and contents of olefins, furans and phenols were higher. Among them, the compounds with major contributions in the protease combined with heat treatment were ionone, *β*-damascenone, 3-methyl-1-butanol, alpha-terpineol and limonene, which helped increase the content of terpenoids and enhance the floral and fruit aroma of the wine. Meanwhile, linalool, diethyl succinate, 2-methyl-3-heptanone, butanal diethyl acetal, hexanal and n-octanol were six compounds with high content of aromatic compounds unique to liqueur wines after protease combined with heat treatment. The sensory evaluation results were consistent with the results of aromatic compound detection, and the overall quality was better. The results may provide a reference for further exploration of protease-based clarifiers suitable for liqueur wines.

## 1. Introduction

Liqueur is a kind of sweet wine with low alcohol that has been fermented and blended [1]. The appropriate combination of alcoholic content, the amount of sugar and volatile compounds, giving liqueur a unique flavor and richness, may play a key role in the acceptance of liqueur by consumers [2,3,4]. The quality and aromatic characteristics of liqueur wines are influenced by various aspects such as grape variety, cultivation, vinification and aging processes [5,6]. Clarity and stability are important parameters to measure the quality of liqueur wines [7]. However, liqueur wines are extremely high in sugar and contain many macromolecules such as proteins, polyphenols and pectins, which make them prone to instability during storage. The addition of clarifying agents is an important process used to regulate and protect the organoleptic properties of the wine and can improve or solve instability problems caused by protein denaturation or aggregation during aging by removing large molecules, such as unstable proteins [8]. Currently, bentonite is a common clarifying agent in wine production due to its low price and good removal of large molecules such as proteins [9]. Numerous studies have reported that bentonite, due to its unique negative electrostatic properties, can extensively adsorb positively charged compounds, affecting the aroma and flavor characteristics of the wine, and that the poor swelling and settling properties of bentonite can also lead to volume loss and waste generation in the wine [10,11]. Therefore, the development of economical alternative clarifying agents to stabilize wines have become a popular topic of investigation by researchers.

Proteases have shown some effectiveness in improving the stability of wines due to their specific enzymatic effect on proteins. It has been shown that enzyme combined with heat treatment was better than treatment alone due to protein unfolding caused by heat treatment, and that heat treatment did not affect the sensory characteristics of the wine [12]. Subsequently, related scholars treated the protein-rich permeate with targeted heat-binding protease after fractionating the white wine by ultrafiltration, and the results showed that it somewhat mitigated the effects of traditional stabilization on wine aroma and flavor, and the heat stability of the reconstituted wine was significantly improved after heating the wine with the addition of protease [7,13].

In this study, we compared the effects of protease combined with heat treatment and bentonite treatment on the physicochemical index, aroma quality and sensory characteristics of Semillon liqueur wine, and identified the unique aromatic compositions and potential differences in liqueur wine after the two clarification and stabilization treatments, with the aim of providing useful insights and technical support for the clarification and stabilization of liqueur wine.

## 2. Results and Discussion

### 2.1. Chemical Components of Liqueur Wines

Table 1 shows the effect of different treatments on the basic composition of liqueur wines. There was no significant effect of protease combined with heat treatment and bentonite treatment on the total acidity, volatile acidity, and pH content of liqueur wines compared to the blank control. The content of residual sugars increased slightly after the protease combined with heat treatment, while the content of residual sugars did not change significantly after the bentonite treatment compared to the blank control, and the heating treatment did not have a significant effect on the composition of liqueur wines. The bentonite and protease combined with heat treatments significantly reduced the total protein content in liqueur wines, with heating only, bentonite and protease combined with heat treatments reducing the protein content by 10.15%, 17.18% and 16.08%, respectively (*p* < 0.05). This indicated that protease combined with heat treatment had less effect of composition in liqueur wines, in agreement with previous research that found this innovative approach to protein stabilization had no significant impact on wine quality [14].

### 2.2. Aroma Quality of Liqueur Wines

#### 2.2.1. Volatile Compound Analysis

The chemical class, content and sensory characteristics of volatile aroma compounds influenced the complexity of liqueur flavors, and the interaction of clarification stabilization treatments on volatile compounds depended on the properties of the compounds, the physicochemical properties of the clarifying agent and possible interactions with other macromolecules in liqueur wines [15,16]. To analyze the effect of protease combined with heat treatment and bentonite treatment on volatile aroma compounds in liqueur wine, HS-SPME-GC-MS was used to examine the liqueur wine samples subjected to different treatments. The data in Table 2 show that a total of 58 volatile aroma compounds were detected in liqueur wines after three different treatments. Compared with the blank control group (44 species, total 108.705 mg/L), 52 species (83.233 mg/L) and 50 species (120.655 mg/L) of aroma compounds were detected in the bentonite and protease combined with heat treatments, respectively, indicating that the types and contents of volatile aroma compounds in liqueur wines changed after different treatments, with the bentonite treatment causing the greatest decrease in the total content of aroma compounds, while the protease combined with heat treatment increased the variety and total content of aroma compounds in the wines.

**Figure 1:**
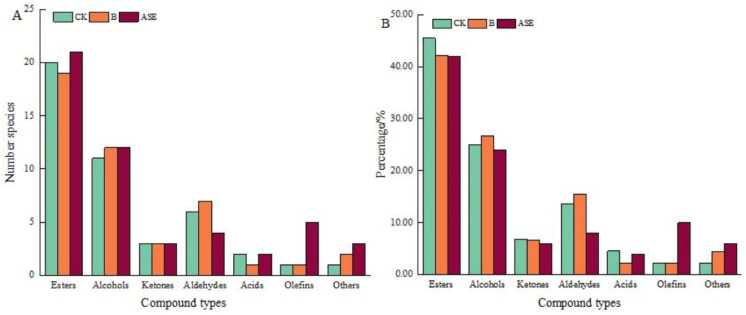
Types and proportion of volatile aroma compounds in liqueur wines after different treatments. CK: blank control, B: bentonite treatment, ASE: protease combined with heat treatment.

As can be seen from Table 2 and Figure 1, the largest number and proportion of ester compounds were found in the wines of all treatment groups, followed by alcohols. Among them, the types of aroma compounds in the blank control were ranked as esters (20) > alcohols (11) > aldehydes (6) > ketones (3) > acids (2) > olefins (1) = others (1), and the percentage ranking was esters (45.45%) > alcohols (25.00%) > aldehydes (13.64%) > ketones (6.82%) > acids (4.55%) > olefins (2.27%) = others (2.27%). Bentonite treatment of aroma compounds ranked as esters (19) > alcohols (12) > aldehydes (7) > ketones (3) > other (2) > acids (1) = olefins (1), with the proportion of ester (42.22%) > alcohols (26.67%) > aldehydes (15.56%) > ketones (6.67%) > other (4.44%) > acids (2.22%) = olefins (2.22%). The aroma compounds in the samples of the protease combined with heat treatment were ranked as esters (21) > alcohols (12) > olefins (5) > aldehydes (4) > ketones (3) = other (3) > acids (2), with the proportion of esters (42.00%) > alcohols (24.00%) > olefins (10.00%) > aldehydes (8.00%) > ketones (6.00%) = others (6.00%) > acids (4.00%).

##### Esters

Esters are among the most important volatile compounds in wines. They are mainly produced by yeast during alcoholic fermentation, are synthetic secretions of yeast cells and are the main source of fruity aroma in liqueur wines [17]. The content of esters in wines generally did not exceed 100 mg/L, but the low threshold of most esters and the interactions between the aromatic compounds made it possible for changes in ester content to also have some effect on the overall aroma of liqueur wines. A total of 22 esters were found in the test wines (Table 2), with contents ranging from 26.03% to 37.82% of the total volatile compound content. The total ester content of the blank control group was 35,569.51 μg/L, while the ester content of the liqueur wines after bentonite and protease combined with heat treatment was 31,479.28 μg/L and 31,410.94 μg/L, respectively, which were 11.50% and 11.69% less than the total ester content of the blank control group, respectively.

Ethyl acetate, isoamyl acetate, ethyl caproate and ethyl caprylate are the four types of esters with high ester content in liqueur wines and are also very typical volatile aroma compounds in wines, mainly giving liqueur wines their fruity aroma. The content of ethyl esters was reduced after bentonite treatment except for ethyl octanoate, diethyl succinate, trans-4-decenoate and ethyl laurate, which was consistent with the findings of Vincenzi et al. [18]. Bentonite treatment resulted in significant removal of ethyl esters and fatty acid esters. Overall, the changes in the content of most esters after protease combined with heat treatment were not significantly different from the blank control group, indicating that the effect of this treatment on esters was lower than that of the bentonite treatment group.

##### Alcohols

Alcohols are another important group of volatile compounds in wines, mainly produced by yeast metabolism during wine fermentation, and have a pungent odor [19,20]. The contribution of alcohols to the aroma depends on their concentration in wines. When their concentration is below 300 mg/L, they can provide a pleasant aroma; above this threshold, an irritating aroma will be felt. A total of 16 alcoholic aroma compounds were detected in the test wines, and the total alcohol content in the blank control, bentonite and protease combined with heat treatments were 58,047.00 μg/L, 38,981.47 μg/L and 755,770.72 μg/L, respectively.

As can be seen from Table 2, four volatile compounds, namely isobutanol, isoamyl alcohol, n-hexanol and phenylethyl alcohol, were present in high levels in each wine sample, giving the liqueur wines a floral aroma. Among them, n-hexanol has a grassy flavor, and its high content leads to a raw green flavor in the wine [21]. When compared to the control group, the content of hexanol in the wines after bentonite and protease combined with heat treatment decreased by 25.77% and 8.83%, respectively, which may reduce its negative impact on the aroma of liqueur wines. Phenylethanol has a floral aroma, and when compared to the control group, the bentonite treatment reduced the content of phenylethanol in the wines by 49.60%, while the protease combined with heat treatment increased the content of phenylethanol in the wines by 0.87%. Isoamyl alcohol has a banana taste, and there was no significant difference in its content in the protease combined with heat treatment compared to the control group, but its content was significantly decreased in the bentonite-treated group (*p* < 0.05). In addition, both linalool and *α*-pinoresinol were detected in all treatment groups, which are both major terpenoids in wines, mainly from free aroma or glycosidic aroma precursors that impart floral and fresh aroma to liqueur wines [22]. The bentonite treatment resulted in significantly lower levels of *α*-pinoresinol than the blank control, by 31.08%, while the protease combined with heat treatment resulted in a 56.29% higher content than the blank control. Vincenzi et al. [18] suggested that the mechanism of aroma losses after bentonite treatment may be due to the direct adsorption of the clay. In this experiment, we also confirmed this.

##### Carbonyl Compounds

The concentration of carbonyl compounds in wines is relatively low, but they can contribute to the overall aroma through a synergistic effect [16,17]. Ten carbonyl compounds (three ketones and seven aldehydes) were detected in the liqueur wines (Table 2). After treatments, the ketones in liqueur wines ranged from 411.99 to 598.40 μg/L and the aldehydes from 12,006.38 to 14,233.23 μg/L. Among them, ionone and *β*-damascenone belong to grape terpenoids and C13-norbornenes, respectively, which can give liqueur wines a floral and fruity aroma. The contents of both were reduced after bentonite treatment, and the protease combined with heat treatment increased the content of both by 47.97% and 90.40%, respectively. Among the aldehydes, the content of all aldehydes decreased after the bentonite or protease combined with heat treatment, except for the content of isobutyraldehyde diethyl acetal, but the effect of protease combined with heat treatment on various aldehydes was less than that of the bentonite treatment.

##### Other Volatile Compounds

Small amounts of organic acids have a positive effect on improving the flavor of liqueur wines, but too high levels can also produce unpleasant sensations [17,19]. Both bentonite and protease combined with heat treatment decreased the content of acetic and octanoic acids in liqueur wines, and protease combined with heat treatment reduced the content of both by 14.38% and 28.29% compared to the control group.

In addition, five olefins, two furans and one phenol were detected in the tested liqueur samples, and the total contents of each substance ranged from 39.44 to 270.27 μg/L, 193.81 to 211.68 μg/L and 0 to 40.29 μg/L, respectively. The protease combined with heat treatment significantly increased the types and contents of olefins, furans and phenols, which contributed to the formation of floral, lemon and almond aroma in liqueur wines.

#### 2.2.2. Principal Component Analysis

**Figure 2:**
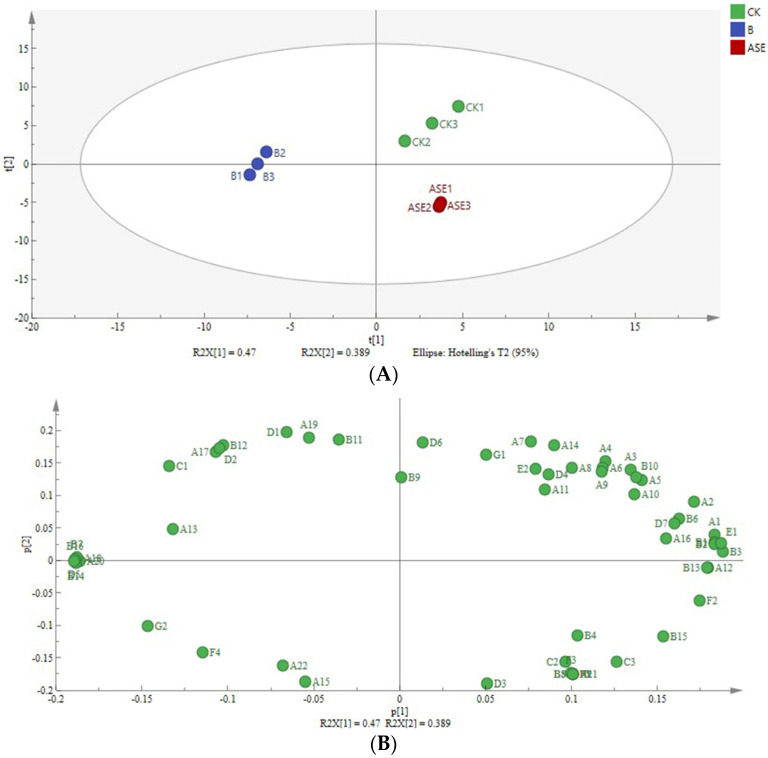
Sample distribution (**A**) and factor loading (**B**) of aroma compound PCA. A_1_-G_3_ in the figure is equivalent to the first column number in Table 2, indicating the corresponding individual volatile compounds.

In general, a variety of volatile aroma compounds were detected in wine, and the content varied greatly. To more directly compare the effects of bentonite and protease combined with heat treatment on the aroma characteristics in liqueur wines, a principal component analysis was performed on the aroma data in Table 2, and the contributions of PC1 and PC2 were 47.00% and 38.90%, respectively. The cumulative variance contribution of the two principal components was 85.90%, which was greater than 80%, i.e., these two principal components could better reflect all of the variance of the original data (Figure 2) [5].

As can be seen from Figure 2, the aroma characteristics of liqueur wine samples from each treatment group can be well distinguished in the two-dimensional plane formed by the two principal components, among which the control group had higher scores in the positive half-axis of PC1 and PC2, which mainly reflected the aroma information of fatty acid ethyl esters, so the floral and fruity characteristics of the control wine samples were more prominent [23]. The wine like aroma profile of the protease combined with heat treatment group scored similar to the control group in the positive half-axis of PC1, while the PC2 score tended to the negative half-axis, with the prominent aroma profile in this region being floral as well as weakly fruity. The bentonite treatment was distributed in the negative half-axis region of PC1 and scored lower in PC2, where the most prominent aromatic characteristics were greasy and slightly fruity. Overall, compared to the bentonite treatment, the protease combined with heat treatment had less effect on the aroma compounds of liqueur wines, and the compounds with major contributions were ionone (violet aroma), *β*-damascenone (floral, fruit, honey), 3-methyl-1-butanol (apple brandy), alpha-terpineol (light, woody aroma) and limonene (lemon aroma), which helped improve terpene aroma in liqueur wines and enhance the floral and fruit aroma of the wine [24].

#### 2.2.3. OPLS-DA Analysis

##### OPLS-DA Modeling and Model Evaluation

The OPLS-DA analysis can effectively distinguish the liqueur wines after different treatments. R^2^X and R^2^Y in the OPLS-DA model indicated the explanation rate of the proposed model for X and Y matrices, respectively, and Q^2^ indicated the predictive ability of the model. Theoretically, the closer R^2^ and Q^2^ are to 1.0, the better the model and the higher the accuracy of the model fit [25]. Usually, R^2^ and Q^2^ higher than 0.5 model is better, higher than 0.4 is acceptable, and the difference between the two should not be too large [26,27]. In this experiment, the model had R^2^X = 0.942, R^2^Y = 0.996 and Q^2^ = 0.986. where R^2^X = 0.942, indicating that the model captures 94.2% of the variation in the data, and R^2^ and Q^2^ were close to 1.0, indicating that the model had good interpretability and a good fit. The samples of each treatment clustered well on the scatter plot of OPLS-DA scores, with small intra-group differences, and complete separation of samples between different groups was achieved.

**Figure 3:**
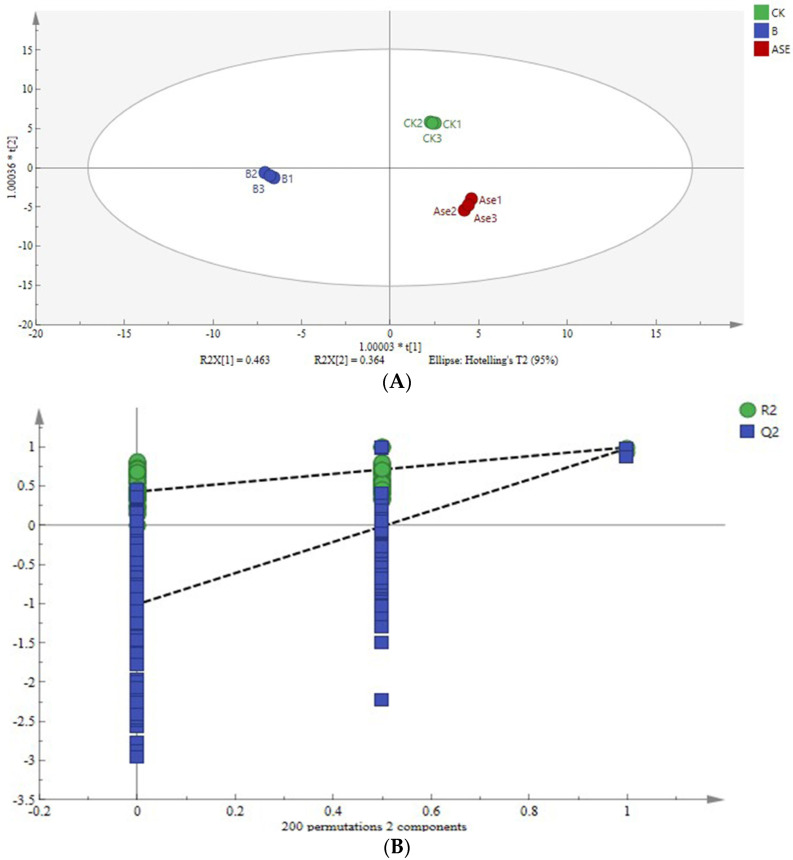
OPLS₋DA analysis of volatile aroma compounds in liqueur wines after different treatments (**A**) and 200 substitution test results (**B**).

In order to avoid the overfitting phenomenon when the OPLS-DA model can effectively differentiate samples between groups but cannot effectively predict the new sample dataset, the permutation test and CV-ANOVA function in SIMCA 14.1 was used to verify the reliability of the model, and the results of the permutation test are shown in Figure 3B. The points at the retention level equal to 1.0 were the R^2^ and Q^2^ of the original OPLS-DA model. During the permutation test, if all R^2^ and Q^2^ are below the value of permutation retention equal to 1.0, and the regression line at Q^2^ crosses the horizontal coordinate or is less than 0, the intercept is generally considered negative and the statistical model is valid and not overfitted [28]. As shown in Figure 3B, after 200 cross-validations, the model Q^2^ regression line still crossed the horizontal coordinate and the intercept of the cross with the vertical coordinate was less than 0. This indicated that the model was not overfitted and the OPLS-DA model established in this study was stable, reliable and statistically significant.

##### Potential Markers of Variability

**Figure 4:**
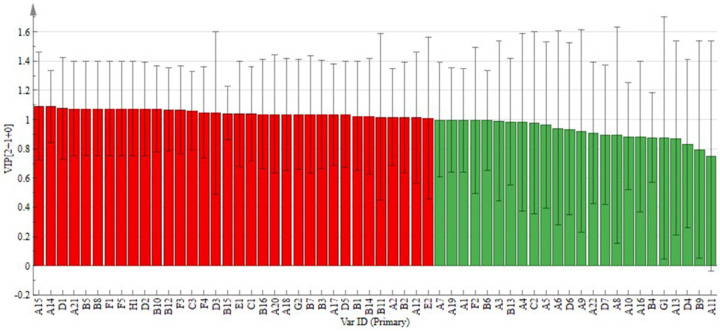
VIP values of volatile aroma compounds in liqueur wines after different treatments. A_1_-G_3_ in the figure is equivalent to the first column number in Table 2, indicating the corresponding individual volatile compounds.

VIP (variable importance in projection) is the weight value of OPLS-DA model variables, which can be used to measure the intensity and explanatory power of the accumulated differences of each component on the classification discrimination of each group of samples; the larger the VIP value, the greater is the contribution [29,30]. Usually, VIP > 1 is the common differential metabolite screening criterion [31]. As shown in Figure 4, there were 34 compounds with VIP > 1, indicating that there were more differential markers in liqueur wines after different treatments, and the type of clarifying agent had a greater effect on volatile aroma compounds in liqueur wines.

##### Hierarchical Clustering Analysis of Sign Variability Components Based on OPLS-DA Model VIP > 1

**Figure 5:**
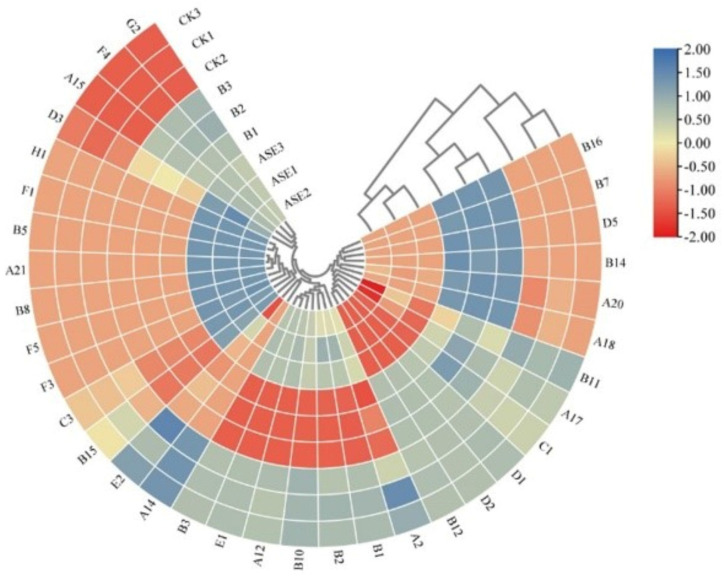
Heat map of significative differential aroma compounds in liqueur wines with VIP > 1 after different treatments. A_1_–G_3_ in the figure is equivalent to the first column number in Table 2, indicating the corresponding individual volatile compounds.

To further analyze the changes in the differential volatile aroma components in liqueur wines after different treatments, a heat map hierarchical clustering analysis was done on 34 marker differential compounds based on Section “Potential Markers of Variability” in order to better represent the trends of 34 differential compounds in different liqueur wines. As shown in Figure 5, the differential aroma compounds in the liqueur wine samples could be divided into two major groups (six subgroups) by hierarchical cluster analysis. The first group was dominated by the co-occurrence of aroma compounds in the protease combined with heat treatment and control liqueur wine samples, and the second group was dominated by the co-occurrence of aroma compounds in the bentonite treatment and control liqueur wine samples. Among them, within the first major category, 12 compounds were the main aroma compounds of the protease combined with heat treatment group and 6 were the main aroma compounds of the control group; in the second major category, 2 compounds were the main aroma compounds of the protease combined with heat treatment group, 18 were the main aroma compounds of the bentonite treatment group, and 13 were the main aroma compounds of the control group. Previous studies have shown some differences in the effects of bentonite treatment and protease combined with heat treatment on the aroma composition of wines, with the protease combined with heat treatment inducing greater modification of aroma compounds (possibly related to the action of protease) [32]. The present study found linalool, diethyl succinate, 2-methyl-3-heptanone, butanal diethyl acetal, hexanal and n-octanol, six compounds with high content of aromatic compounds unique to liqueur wines after protease combined with heat treatment. Seven compounds, namely ethyl acetate, propanol, isobutanol, 2,3-butanediol, methyl dimethoxyacetate, acetic acid and n-butanol, were the aroma compounds with high contents specific to the bentonite treatment. Ethyl trans-2-hexenoate, styrene and 3,6-dihydro-4-methyl-2-(2-methyl-1-propenyl)-2*H*-pyran were the aroma compounds with high contents that were unique to the control samples.

### 2.3. Sensory Evaluation

**Figure 6:**
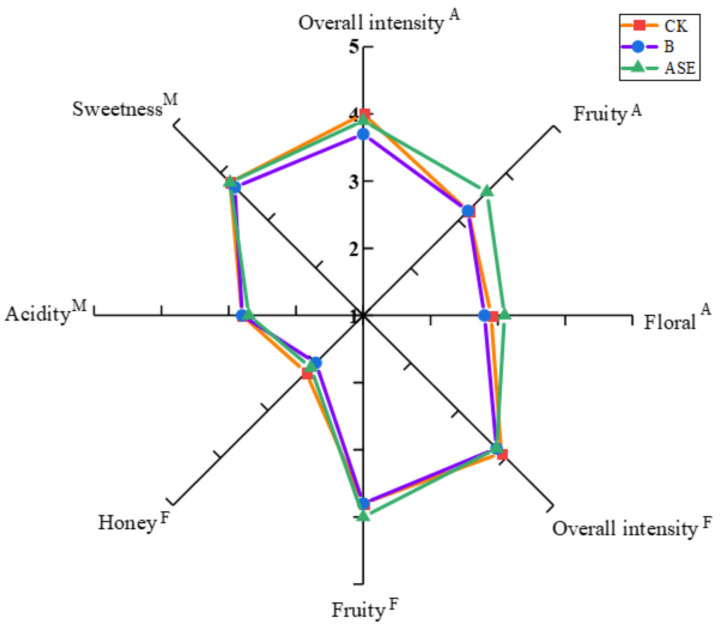
Radar plot of sensory evaluation of wine samples after different treatments.

As shown in Figure 6, after sensory evaluation by the evaluators, it was found that among a total of 18 sensory attributes involving aroma, flavor and mouthfeel in the test, the liqueur wines presented after different treatments had high ratings for eight sensory attributes, including overall intensity (A), fruitiness (A), floral (A), overall intensity (F), fruitiness (F), honey (F), acidity (M) and sweetness (M), where the overall aroma intensity scores were similar among all the wines, and the overall odor and flavor intensity of the different treated wine samples had high ratings, indicating that the liqueur wines had better flavor characteristics. In addition, fruity, floral and sweetness were the most prominent characteristics of the liqueur wine samples, which was consistent with the results of the analysis of the volatile matter data of the liqueur wine samples from the different treatments in the previous part of this study. Some (but not all) volatile alcohols, esters and terpenes imparted desirable fruity and floral aroma [33] and these volatiles were abundant in ASE. Thus, compared to the control and bentonite treatments, the protease combined with heat treatment was more prominent in terms of fruit (A) and floral (A) odors. Meanwhile, the distinctive organoleptic characteristics of liqueurs were usually closely related to their high sugar content, while the oxidation, Maillard reaction, browning and caramelization reactions that occurred during the aging phase may also give fortified wines their unique organoleptic characteristics [34].

## 3. Materials and Methods

### 3.1. Materials and Chemicals

Semillon grapes (24.6 °Brix) were harvested in 2020 from wine-growing sites in Qilian, Gaotai County, Zhangye City, Gansu Province, China; ugni blanc brandy (51% vol) was sourced from the Gansu Province Wine Industry Technology Research and Development Center, China; and Italian Riesling ice wine (9% vol) was obtained from Qilian Wine Co., Zhangye City, Gansu Province, China.

AROMA White active yeast (*Saccharomyces cerevisiae*) was purchased from Enartis, Italy, and acidic protease, derived from *Aspergillus niger*, 50 U/mg, was purchased from Shanghai Yuanye Biotechnology Co. (Shanghai, China).

### 3.2. Vinifications and Samples

#### 3.2.1. Small-Scale Winemaking

Grapes of uniform ripeness were selected, destemmed and crushed, and 60 mg/L SO_2_ (as sodium sulfite) and 35 mg/L pectinase were added uniformly to the must, which was rapidly cooled down and left to stand at 10 °C for 8 h. After clarification, the precipitate was separated, and 600 mg/L bentonite was added and left to stand for 4 d at 2 °C. Subsequently, the precipitation was separated again. Alcoholic fermentation was carried out at 20 ± 1 °C with 0.2 g/L addition of yeast, and the fermented base wine was stored at 14~16 °C for 1~2 months after the fermentation.

#### 3.2.2. Liqueur Preparation

The Semillon liqueur wine with 18.8% vol alcohol and 98.90 g/L total sugar was formulated with Semillon white wine (30.5%, *v*/*v*), ugni blanc brandy (27.8%, *v*/*v*), Italian Riesling ice wine (12.6%, *v*/*v*), and Semillon grape juice (29.1%, *v*/*v*) with reference to the blending parameters optimized by Ding et al. [35].

#### 3.2.3. Clarifying Treatment

Pre-tests were conducted to determine the minimum bentonite dose and the optimal amount of protease addition required for liqueur wine stabilization. Specifically, treatment groups were: (H) treated with heat only (50 °C, 3 h); (B) 0.3 g/L bentonite added in the form of aqueous suspension (2% *W*/*V*); and (ASE) 0.4 g/L protease added and kept at a constant temperature of 50 °C for 3 h. The treated liqueur wine samples were stored at 20 °C for 48 h. The sediment was separated and the clarified liquid was collected for analysis.

### 3.3. Chemical Analysis

Liqueur composition, including residual sugar, total acid, volatile acid and pH, was determined using a multifunctional wine analyzer [36].

The protein content was determined by using the Coomassie brilliant blue G-250 method with reference to the method of Bradford et al. [37]. Specifically, 1 mL of the wine sample to be tested was aspirated, 5 mL of Coomassie brilliant blue G250 reagent was added, shaken thoroughly and mixed well, and then allowed to rest for 2 min. Absorbance was measured at 595 nm, and a standard curve was obtained using bovine serum albumin as the standard.

### 3.4. Volatile Analysis

Extraction and analysis of volatile compounds followed the methods proposed by Ma et al. [5].

#### 3.4.1. Aroma Enrichment

For headspace solid-phase microextraction (HS-SPME), 8 mL of wine sample was taken in a 15 mL headspace vial, and 2.4 g of NaCl and 20 µL of the internal standard 2-octanol (concentration 82.07 mg/L) were added, sealed with a rotor, placed on a magnetic stirrer, equilibrated in a water bath at 40 °C for 30 min and then extracted in headspace for 30 min. After the extraction, the extraction head was removed and inserted into a GC-MS coupler and aroma detection was performed.

#### 3.4.2. GC-MS Conditions

Chromatographic conditions: chromatographic column: DB-WAX (60 m × 2.5 mm × 0.25 μm); ramp-up procedure: 40 °C for 7 min, 4 °C/min to 200 °C for 8 min; carrier gas (He) flow rate 1 mL/min; injection port temperature 240 °C; no split injection.

Mass spectrometry conditions: electron bombardment ion source (EI); electron energy 70 eV; transmission line temperature 220 °C; ion source temperature 240 °C; mass spectrometry scan range *m*/*z* 50 to 350.

#### 3.4.3. Volatile Composition Analysis

Qualitative analysis: retention index (RI) and NIST-11, Wiley and flavor and fragrance library search comparison were used for characterization, and a match of >700 was required for library comparison.

Quantitative analysis: the concentration of volatile aroma compounds was carried out by the internal standard method with 2-octanol as the internal standard. The calculation formula was as follows:

$$X=A_{1}\times C/A$$

where X indicates mass concentration of the aroma substance, µg/L; A_1_ is the peak area of the measured aroma substance; C is the mass concentration of the internal standard, µg/L; and A is the peak area of the measured internal standard.

### 3.5. Sensory Analysis

The sensory characteristics of liqueur wines were performed by a group of non-expert consumers using the rate-all-that-apply (RATA) sensory method [38]. The sensory descriptors evaluated in Table 3 were determined by referring to the method of Sui et al. [14] and incorporating the sensory attributes of the grape varieties used in liqueurs. Specifically, the panel members (n = 20, 10 females and 10 males, aged 18~35 years) were wine professionals from the College of Food Science and Engineering, Gansu Agricultural University with extensive knowledge of wine tasting. The sensory attributes of the corresponding grape varieties and the use of the RATA procedure were explained to the panelists before the start of the tasting. The RATA evaluation was carried out with controlled environmental conditions (i.e., lighting and at a constant 22 ± 1 °C). During tasting, chilled liqueur wine samples (10 °C, 25 mL) were served in 4-digit coded, clear 215 mL glasses using a randomized presentation order. Panelists assessed the intensity of each sensory attribute, where 0 = “not perceived”, 1 = “extremely low”, 4 = “moderate” and 7 = “extremely high”. A 1 min break was taken between samples, one sample at a time, and water and plain crackers were provided for epiglottal cleansing.

### 3.6. Statistical Analysis

All tests were repeated three times, plotted using Origin 2018, SIMCA 14.1 and TBtools software, and data were statistically analyzed by SPSS 22. Analysis of variance (ANOVA) was used to analyze the data of all physicochemical parameters. Tukey’s range test was used to determine the significant differences at *p* < 0.05, and data are expressed as mean ± standard deviation.

## 4. Conclusions

The protease combined with heat treatment had a certain stabilizing effect on proteins, and had no significant negative effect on other nutrients of liqueurs. A total of 58 volatile compounds were detected by HS-SPME-GC/MS. Compared with the blank control (44 species, total 108.705 mg/L), 52 (83.233 mg/L) and 50 (120.655 mg/L) compounds were detected in the bentonite and protease combined with heat treatments, respectively. The protease combined with heat treatment helped to improve the terpene aroma and enhance the floral and fruity aroma in the liqueurs, and the compounds with major contributions were mainly ionone, *β*-damascenone, 3-methyl-1-butanol, alpha-terpineol and limonene. OPLS-DA achieved an accurate differentiation of the differential aroma compounds in the liqueur wines after different treatments. Therefore, protease combined with heat treatment can be used as an alternative to bentonite for the clarification and stabilization of liqueurs.

## Figures and Tables

**Table 1:** Effect of different treatments on the basic composition in liqueur wines.

	Residual Sugar/(g/L)	Total Acidity/(g/L)	Volatile Acidity/(g/L)	pH	Protein/(mg/L)
CK	98. 90 ± 0.75 ^bc^	4.24 ± 0.07 ^a^	0.57 ± 0.00 ^a^	4.11 ± 0.01 ^a^	59.86 ± 0.99 ^a^
H	99.23 ± 1.75 ^ab^	4.23 ± 0.06 ^a^	0.57 ± 0.02 ^a^	4.11 ± 0.00 ^a^	53.60 ± 0.25 ^b^
B	96.97 ± 0.06 ^c^	4.17 ± 0.01 ^a^	0.56 ± 0.01 ^a^	4.11 ± 0.00 ^a^	49.57 ± 0.98 ^c^
ASE	101.10 ± 0.90 ^a^	4.20 ± 0.04 ^a^	0.56 ± 0.02 ^a^	4.11 ± 0.00 ^a^	50.30 ± 0.25 ^c^

CK: blank control, H: heat treatment only, B: bentonite treatment, ASE: protease combined with heat treatment. Different lowercase letters in the table indicate significant differences among treatments (*p* < 0.05).

**Table 2:** Types and concentrations of aroma compounds in liqueur wines after different treatments.

Number	Compound Name	Average Content (µG·L^−1^)			Odor Description
		CK	B	ASE	
**Esters**					
A1	Ethanedioic acid, diethyl ester	142.90 ± 30.81 ^a^	88.55 ± 11.68 ^b^	137.46 ± 1.49 ^a^	/
A2	Ethyl acetate	14,853.45 ± 3048.37 ^a^	8272.05 ± 753.22 ^b^	12,345.61 ± 505.73 ^a^	Banana, strawberry fragrance, fruit flavor
A3	Ethyl propionate	323.53 ± 71.67 ^a^	225.61 ± 17.29 ^b^	259.05 ± 3.79 ^b^	Fruity
A4	Ethyl isobutyrate	123.78 ± 22.57 ^a^	95.71 ± 11.03 ^b^	102.68 ± 1.57 ^b^	Apple, floral, rubber, strawberry, sweet
A5	Isobutyl acetate	99.48 ± 22.09 ^a^	72.5 ± 10.49 ^b^	84.47 ± 0.73 ^ab^	Sweet, banana, fruity, fruity aroma
A6	Ethyl butanoate	503.95 ± 114.94 ^a^	389.33 ± 55.90 ^b^	422.52 ± 9.31 ^ab^	Papaya, creamy fragrance, pineapple, strawberry flavor
A7	Ethyl 2-methylbutanoate	47.03 ± 7.21 ^a^	37.06 ± 3.14 ^b^	35.96 ± 3.60 ^b^	Fennel, apple, bubblegum, fruit, kiwi
A8	Ethyl isovalerate	56.51 ± 12.89 ^a^	46.45 ± 7.79 ^a^	48.62 ± 2.36 ^a^	Fennel, apple, citrus, fruit, pineapple
A9	Isoamyl acetate	2143.51 ± 517.38 ^a^	1675.69 ± 230.34 ^a^	1823.19 ± 54.54 ^a^	Sweet fruit, banana, green apple
A10	Ethyl valerate	117.48 ± 22.51 ^a^	82.75 ± 6.53 ^b^	100.31 ± 26.84 ^ab^	Apple, dried fish, herbs, nuts, yeast
A11	Ethyl hexanoate	4385.23 ± 1064.73 ^a^	3828.77 ± 55.38 ^a^	3962.73 ± 585.16 ^a^	Fruity, strawberry, pineapple, banana fragrance, green apple
A12	Methyl 2,2-dimethoxyacetate	110.88 ± 21.23	nd	114.01 ± 35.48	/
A13	Ethyl caprylate	10,283.06 ± 2073.98 ^b^	12,903.69 ± 1777.82 ^a^	10,020.47 ± 1562.42 ^b^	Fruity, oily, fruity, ripe fruit, pear, sweet aroma
A14	Ethyl orthoformate	311.89 ± 27.09	nd	nd	/
A15	Hex-(2 E)-enoate <ethyl->	nd	26.26 ± 2.84	32.63 ± 0.00	/
A16	Ethyl furoate	143.31 ± 22.92 ^a^	121.86 ± 12.36 ^a^	143.41 ± 12.62 ^a^	Floral fragrance
A17	Diethyl succinate	589.97 ± 28.38 ^a^	615.95 ± 67.93 ^a^	403.49 ± 63.70 ^b^	Almond fragrance
A18	Ethyl trans-4-decenoate	181.54 ± 38.26 ^b^	1158.42 ± 43.22 ^a^	186.22 ± 4.99 ^b^	/
A19	Phenethyl acetate	840.25 ± 142.26 ^a^	807.7 ± 114.43 ^a^	612.99 ± 36.36 ^b^	Rose, jasmine aroma, floral, sweet
A20	Dodecanoate <ethyl->	210.21 ± 34.08 ^b^	735.59 ± 40.59 ^a^	227.19 ± 27.57 ^b^	Sweet, beeswax, floral and fruity
A21	Benzoic acid, 2-methylpropyl ester	nd	nd	40.35 ± 0.00	/
A22	Whiskey lactone	101.54 ± 12.08 ^b^	295.35 ± 202.81 ^a^	307.58 ± 33.59 ^a^	Citrus flavor, coconut flavor
Total		35,569.51 ± 7335.47	31,479.28 ± 3424.82	31,410.94 ± 2971.84	
**Alcohols**					
B1	1-Propanol	792.08 ± 37.30 ^a^	288.97 ± 5.14 ^b^	720.82 ± 150.20 ^a^	Vegetable Aroma
B2	Isobutyl alcohol	3283.03 ± 231.54^a^	1240.1 ± 8.25 ^b^	3051.51 ± 712.14 ^a^	Solvent taste, raw green flavor
B3	1-Butanol	71.81 ± 5.39	nd	67.25 ± 12.02	Herbal, alcoholic odor
B4	3-Methyl-1-butanol	47,175.56 ± 31,546.15 ^ab^	33,588.39 ± 904.38 ^b^	65,588.85 ± 12,669.62 ^a^	Apple brandy, spicy
B5	1-Pentanol	nd	nd	48.61 ± 0.00	Spicy, grassy aroma
B6	1-Hexanol	1648.18 ± 25.97 ^a^	1223.39 ± 50.94 ^b^	1502.62 ± 208.01 ^a^	Herbaceous, grassy fragrance
B7	3-Hexen-1-ol	nd	27.23 ± 3.20	nd	Floral, botanical, fruity
B8	3-Hexen-1-ol, (Z)-	nd	nd	42.42 ± 0.00	Grass flavor
B9	1-Heptanol	50.46 ± 0.66 ^a^	47.01 ± 2.35 ^a^	44.31 ± 8.17 ^a^	Grape flavor
B10	Butadienol <2,3->	469.36 ± 23.64	nd	158.26 ± 0.00	Buttery, creamy, rubbery, fruity
B11	Linalool	272.86 ± 5.10 ^a^	252.08 ± 20.08 ^a^	212.94 ± 30.04 ^b^	Floral, lavender fragrance
B12	1-Octanol	68.52 ± 8.69	71.32 ± 5.34	nd	Stimulating aromatic scent, jasmine, lemon
B13	Phenylethyl alcohol	4068.14 ± 82.68 ^a^	2050.14 ± 356.61 ^b^	4103.36 ± 788.08 ^a^	Rose, floral, sweet fragrance
B14	2-Hexadecanol	nd	31.95 ± 7.07	nd	/
B15	alpha-Terpineol	147.02 ± 30.82 ^b^	101.32 ± 1.21 ^c^	229.78 ± 8.40 ^a^	Fresh, woody fragrance
B16	Triethylene glycol monododecyl ether	nd	59.59 ± 2.12	nd	/
Total		58,047.00 ± 31,997.94	38,981.47 ± 1366.72	75,770.72 ± 14,586.69	
**Ketones**					
C1	2-methyl-3-Heptanone	51.07 ± 0.75 ^b^	58.19 ± 8.05 ^a^	32.11 ± 0.00 ^c^	/
C2	Ionone	335.52 ± 5.52 ^b^	324.53 ± 9.59 ^b^	496.47 ± 119.79 ^a^	Violet fragrance
C3	Damascenone <(E)-, beta->	36.67 ± 1.28 ^b^	29.26 ± 0.00 ^c^	69.82 ± 1.41 ^a^	Poached apples, floral scents, fruits, honey
Total		423.27 ± 7.55	411.99 ± 17.64	598.40 ± 121.20	
**Aldehydes**					
D1	1,1-Diethoxybutane	521.59 ± 99.38	425.24 ± 58.79	nd	/
D2	Hexanal	33.23 ± 1.07	34.16 ± 1.01	nd	Grassy smell, apple fragrance
D3	Isobutyraldehyde Diethyl Acetal	153.69 ± 14.28 ^c^	226.07 ± 17.68 ^b^	408.07 ± 68.99 ^a^	/
D4	Furfural	11,446.78 ± 2760.11 ^a^	9599.19 ± 1636.49 ^a^	9902.16 ± 1327.07 ^a^	Herbal, tea, almond flavor, floral
D5	Methylal	nd	57.83 ± 5.78	nd	/
D6	Benzaldehyde	438.71 ± 4.24 ^a^	387.21 ± 61.49 ^ab^	351.37 ± 31.49 ^b^	Bitter almond flavor, oily flavor
D7	5-Methyl furfural	1639.23 ± 251.23 ^a^	1276.68 ± 104.18 ^b^	1560.78 ± 226.86 ^ab^	Caramel smell
Total		14,233.23 ± 3130.31	12,006.38 ± 1885.42	12,222.38 ± 1654.41	
**Acids**					
E1	Acetic acid	82.07 ± 2.95	nd	70.27 ± 0.00	Acetic acid smell
E2	Octanoic acid	109.19 ± 12.73 ^a^	76.45 ± 4.39 ^b^	78.3 ± 19.66 ^b^	Fatty acids, dairy products
Total		191.26 ± 15.68	76.45 ± 4.39	148.58 ± 19.66	
**Olefins**					
F1	alpha-Pinene	nd	nd	47.39 ± 0.00	Pine wood, resin incense
F2	γ-Terpinene	39.44 ± 4.24	nd	60.03 ± 22.26	Bitter, citrus, gasoline, resin, turpentine
F3	Limonene	nd	nd	60.26 ± 13.14	Lemon scent
F4	Styrene	nd	56.1 ± 13.05	44.72 ± 7.31	Special fragrance
F5	Octene <alpha->	nd	nd	57.87 ± 0.00	/
Total		39.44 ± 4.24	56.10 ± 13.05	270.27 ± 42.71	
**Others**					
G1	Bois de Rose oxide	200.84 ± 48.69 ^a^	176.72 ± 27.46 ^a^	168.67 ± 7.62 ^a^	/
G2	Nerol oxide	nd	44.96 ± 13.28	25.14 ± 0.00	Flowers, oil
G3	Butylated Hydroxytoluene	nd	nd	40.29 ± 0.00	Toasted bread slices
Total		200.84 ± 48.69	211.68 ± 40.74	234.10 ± 7.62	

“nd” indicates that the aroma compounds were not detected or the content was very small, “/” indicates that the relevant information was not found. Different lowercase letters in the table indicated significant differences among treatments (*p* < 0.05). Treatment abbreviations as in Table 1.

**Table 3:** Table of sensory evaluation attributes.

Aroma (A)	Flavor (F)	Mouthfeel (M)	Sensory Intensity Score
Overall intensity	Overall intensity	Acidity	0 = “ not perceived “ 1 = “ extremely low “ 4 = “ moderate “ 7 = “ extremely high “
Fruity	Fruity	Bitterness	
Floral	Floral	Sweetness	
Honey	Honey	Dryness	
Herbaceous	Herbaceous	Astringency	
Solvent	Solvent	Alcohol heat/warmth	

## Data Availability

The data presented in this study are available within the article.
